# “We weren’t used to seeing our colleagues hospitalized”: A clinical-qualitative study on reports from an intensivist clinical team at a Brazilian university public hospital

**DOI:** 10.1192/j.eurpsy.2023.1663

**Published:** 2023-07-19

**Authors:** E. R. Turato, F. S. Santos, L. M. Guerra, A.-P. D.-C. Gasparotto, R. N. Aoki, J. M. Cavalcante

**Affiliations:** 1 Laboratory of Clinical-Qualitative Research, State University of Campinas, Campinas, Brazil

## Abstract

**Introduction:**

The care relationships of physicians and nurses with patients with Covid-19 had pointed to a scenario explorable from a psychological point of view due to the peculiarities of this pandemic. How do clinicians feel, when caring for their co-workers, in a context that was not so common to see colleagues occupy the patient’s place? What emotional experiences arise from this reality? The results of the present study sought to point out how to handle this caring relationship, in an exceptional context.

**Objectives:**

To interpret emotional meanings reported by physicians and nurses on their experiences of working at COVID-19 intensive care units during the height of the pandemic.

**Methods:**

Clinical-qualitative design of Turato. Data collection with semi-directed interviews with open-ended questions in-depth applied to a sample of six professionals, closed by theoretical information saturation according to Fontanella, in a Brazilian university general hospital. Trigger question: “Talk about the psychological meanings of your experience in face of management of patients with COVID-19 at ICU”. Data treatment by the Seven Steps of the Clinical-Qualitative Content Analysis of Faria-Schützer. Theoretical framework from Medical Psychology using Balintian concepts.

**Results:**

We raised initially 4 categories. Three categories were presented preliminarily in this congress, version last year. In this opportunity, we show this special category of analysis that emerged during the deepened discussion of the final results: “The feeling of insecurity: from technique to affective dimension”.

**Conclusions:**

The care relationships between the health professional and the patient hospitalized in the Covid-19 ICU pointed to peculiar transference and countertransference psychodynamic mechanisms between both. Before the pandemic, the care relationship seemed pragmatic and protocolar. During the pandemic, this relationship seemed “more subjective”, building a strongly emotional dimension, as health professionals also began to care for their colleagues in the profession. The egoic defense mechanisms, such as projective and introjective identification were reported as intense.

**Disclosure of Interest:**

None Declared

